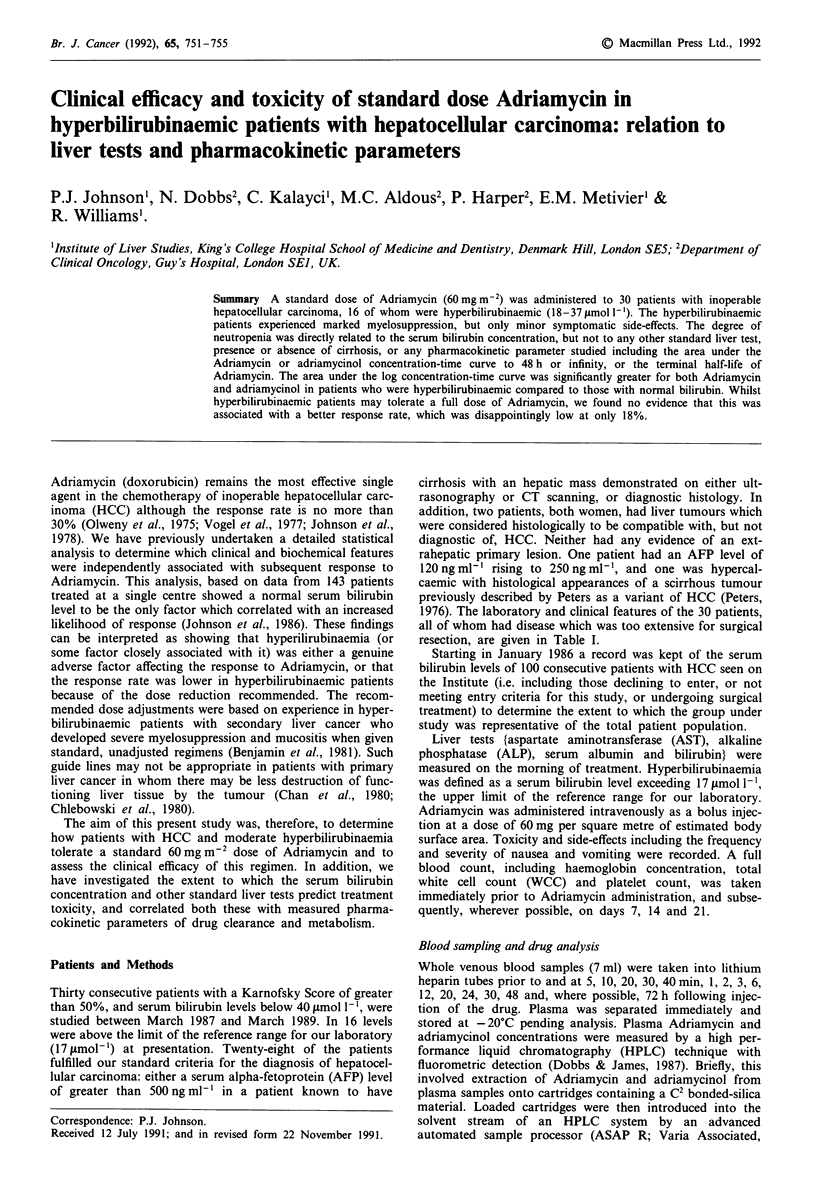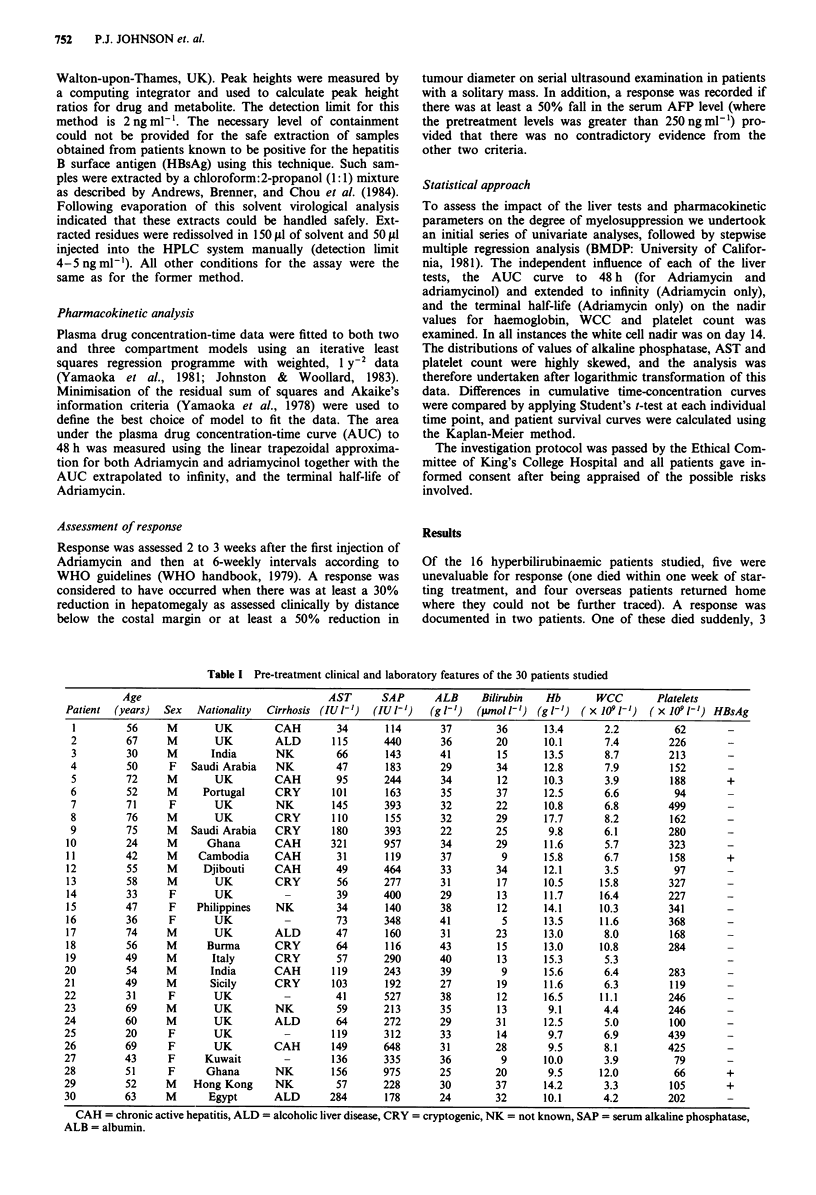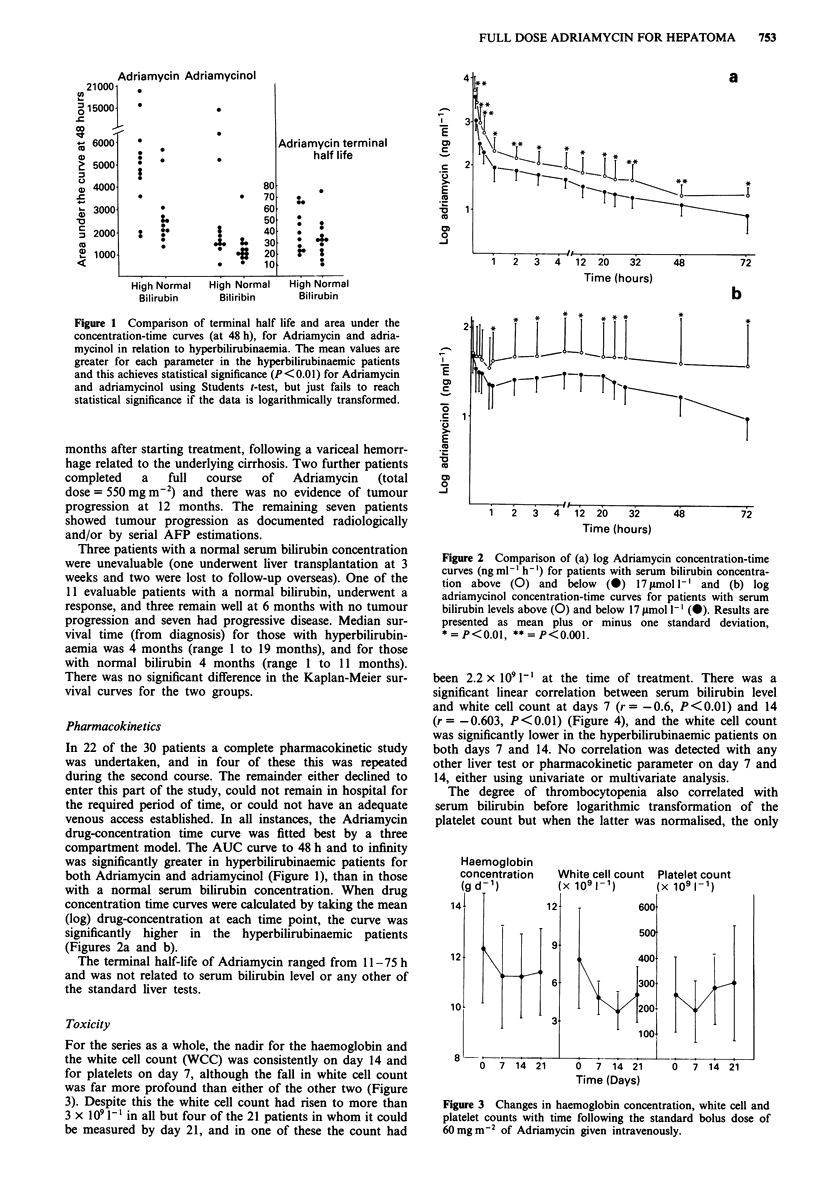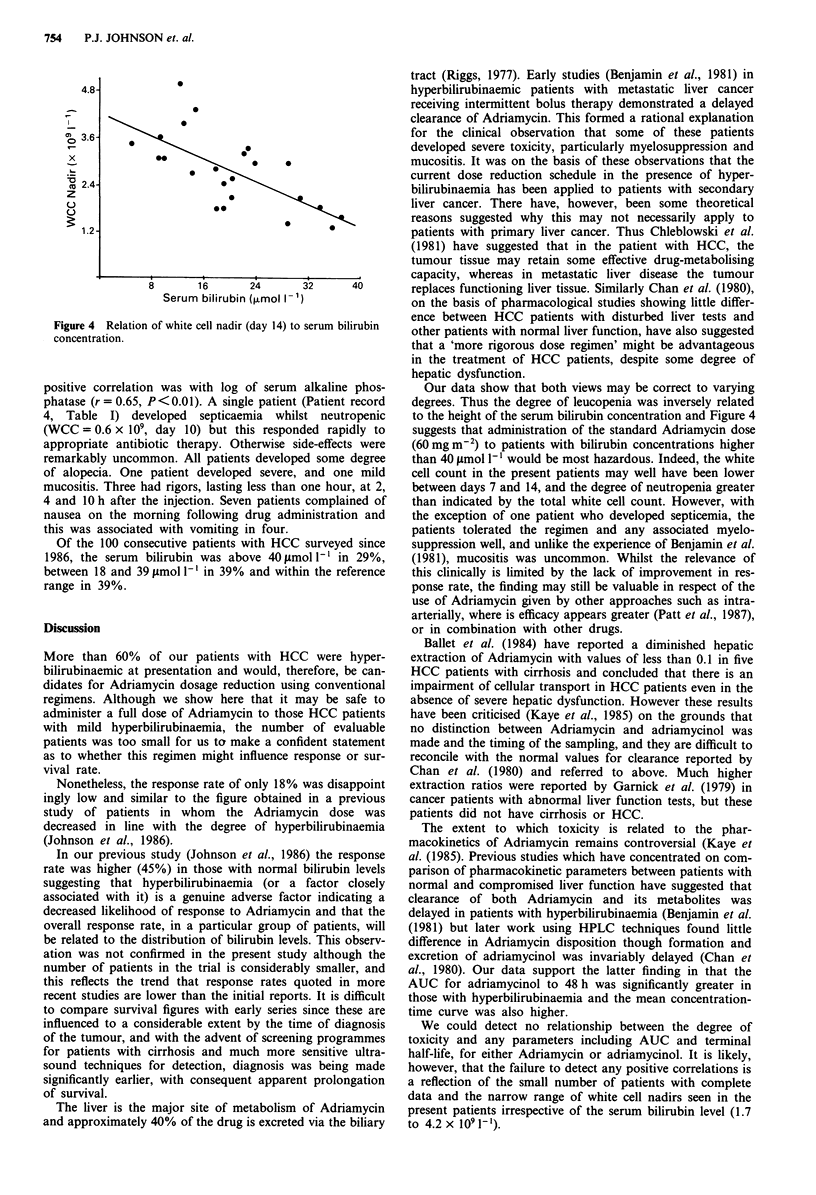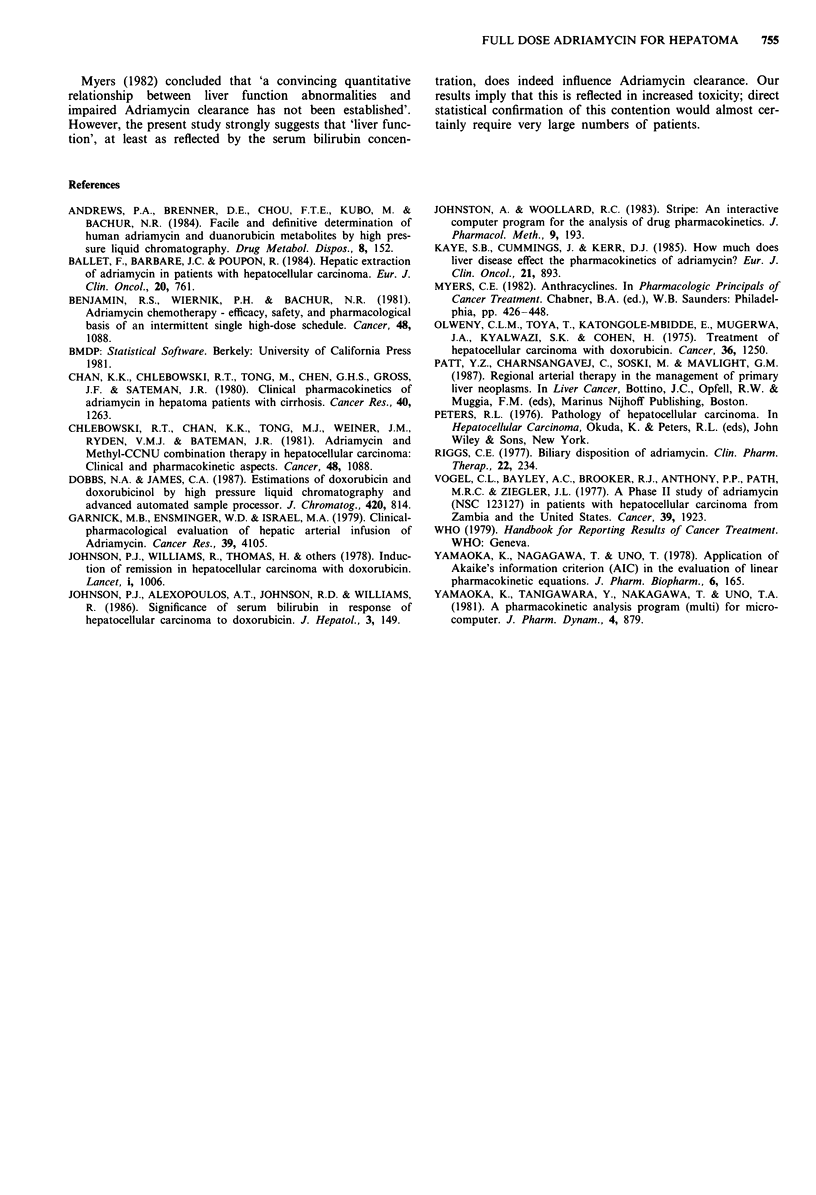# Clinical efficacy and toxicity of standard dose adriamycin in hyperbilirubinaemic patients with hepatocellular carcinoma: relation to liver tests and pharmacokinetic parameters.

**DOI:** 10.1038/bjc.1992.158

**Published:** 1992-05

**Authors:** P. J. Johnson, N. Dobbs, C. Kalayci, M. C. Aldous, P. Harper, E. M. Metivier, R. Williams

**Affiliations:** Institute of Liver Studies, King's College Hospital School of Medicine and Dentistry, London, UK.

## Abstract

A standard dose of Adriamycin (60 mg m-2) was administered to 30 patients with inoperable hepatocellular carcinoma, 16 of whom were hyperbilirubinaemic (18-37 mumol l-1). The hyperbilirubinaemic patients experienced marked myelosuppression, but only minor symptomatic side-effects. The degree of neutropenia was directly related to the serum bilirubin concentration, but not to any other standard liver test, presence or absence of cirrhosis, or any pharmacokinetic parameter studied including the area under the Adriamycin or adriamycinol concentration-time curve to 48 h or infinity, or the terminal half-life of Adriamycin. The area under the log concentration-time curve was significantly greater for both Adriamycin and adriamycinol in patients who were hyperbilirubinaemic compared to those with normal bilirubin. Whilst hyperbilirubinaemic patients may tolerate a full dose of Adriamycin, we found no evidence that this was associated with a better response rate, which was disappointingly low at only 18%.


Br. J. Cancer (1992), 65, 751–755 © Macmillan Press Ltd., 1992

### Clinical efficacy and toxicity of standard dose Adriamycin in hyperbilirubinaemic patients with hepatocellular carcinoma: relation to liver tests and pharmacokinetic parameters

P.J. Johnson[1], N. Dobbs[2], C. Kalayci[1], M.C. Aldous[2], P. Harper[2], E.M. Metivier[1] & R. Williams[1].

[1]*Institute of Liver Studies, King's College Hospital School of Medicine and Dentistry, Denmark Hill, London SE5;* [2]*Department of Clinical Oncology, Guy's Hospital, London SE1, UK.*

**Summary** A standard dose of Adriamycin (60 mg m$^{-2}$) was administered to 30 patients with inoperable hepatocellular carcinoma, 16 of whom were hyperbilirubinaemic (18–37 µmol l$^{-1}$). The hyperbilirubinaemic patients experienced marked myelosuppression, but only minor symptomatic side-effects. The degree of neutropenia was directly related to the serum bilirubin concentration, but not to any other standard liver test, presence or absence of cirrhosis, or any pharmacokinetic parameter studied including the area under the Adriamycin or adriamycinol concentration-time curve to 48 h or infinity, or the terminal half-life of Adriamycin. The area under the log concentration-time curve was significantly greater for both Adriamycin and adriamycinol in patients who were hyperbilirubinaemic compared to those with normal bilirubin. Whilst hyperbilirubinaemic patients may tolerate a full dose of Adriamycin, we found no evidence that this was associated with a better response rate, which was disappointingly low at only 18%.

Adriamycin (doxorubicin) remains the most effective single agent in the chemotherapy of inoperable hepatocellular carcinoma (HCC) although the response rate is no more than 30% (Olweny *et al.*, 1975; Vogel *et al.*, 1977; Johnson *et al.*, 1978). We have previously undertaken a detailed statistical analysis to determine which clinical and biochemical features were independently associated with subsequent response to Adriamycin. This analysis, based on data from 143 patients treated at a single centre showed a normal serum bilirubin level to be the only factor which correlated with an increased likelihood of response (Johnson *et al.*, 1986). These findings can be interpreted as showing that hyperilirubinaemia (or some factor closely associated with it) was either a genuine adverse factor affecting the response to Adriamycin, or that the response rate was lower in hyperbilirubinaemic patients because of the dose reduction recommended. The recommended dose adjustments were based on experience in hyperbilirubinaemic patients with secondary liver cancer who developed severe myelosuppression and mucositis when given standard, unadjusted regimens (Benjamin *et al.*, 1981). Such guide lines may not be appropriate in patients with primary liver cancer in whom there may be less destruction of functioning liver tissue by the tumour (Chan *et al.*, 1980; Chlebowski *et al.*, 1980).

The aim of this present study was, therefore, to determine how patients with HCC and moderate hyperbilirubinaemia tolerate a standard 60 mg m$^{-2}$ dose of Adriamycin and to assess the clinical efficacy of this regimen. In addition, we have investigated the extent to which the serum bilirubin concentration and other standard liver tests predict treatment toxicity, and correlated both these with measured pharmacokinetic parameters of drug clearance and metabolism.

### Patients and Methods

Thirty consecutive patients with a Karnofsky Score of greater than 50%, and serum bilirubin levels below 40 µmol l$^{-1}$, were studied between March 1987 and March 1989. In 16 levels were above the limit of the reference range for our laboratory (17 µmol$^{-1}$) at presentation. Twenty-eight of the patients fulfilled our standard criteria for the diagnosis of hepatocellular carcinoma: either a serum alpha-fetoprotein (AFP) level of greater than 500 ng ml$^{-1}$ in a patient known to have cirrhosis with an hepatic mass demonstrated on either ultrasonography or CT scanning, or diagnostic histology. In addition, two patients, both women, had liver tumours which were considered histologically to be compatible with, but not diagnostic of, HCC. Neither had any evidence of an extrahepatic primary lesion. One patient had an AFP level of 120 ng ml$^{-1}$ rising to 250 ng ml$^{-1}$, and one was hypercalcaemic with histological appearances of a scirrhous tumour previously described by Peters as a variant of HCC (Peters, 1976). The laboratory and clinical features of the 30 patients, all of whom had disease which was too extensive for surgical resection, are given in Table I.

Starting in January 1986 a record was kept of the serum bilirubin levels of 100 consecutive patients with HCC seen on the Institute (i.e. including those declining to enter, or not meeting entry criteria for this study, or undergoing surgical treatment) to determine the extent to which the group under study was representative of the total patient population.

Liver tests {aspartate aminotransferase (AST), alkaline phosphatase (ALP), serum albumin and bilirubin} were measured on the morning of treatment. Hyperbilirubinaemia was defined as a serum bilirubin level exceeding 17 µmol l$^{-1}$, the upper limit of the reference range for our laboratory. Adriamycin was administered intravenously as a bolus injection at a dose of 60 mg per square metre of estimated body surface area. Toxicity and side-effects including the frequency and severity of nausea and vomiting were recorded. A full blood count, including haemoglobin concentration, total white cell count (WCC) and platelet count, was taken immediately prior to Adriamycin administration, and subsequently, wherever possible, on days 7, 14 and 21.

### *Blood sampling and drug analysis*

Whole venous blood samples (7 ml) were taken into lithium heparin tubes prior to and at 5, 10, 20, 30, 40 min, 1, 2, 3, 6, 12, 20, 24, 30, 48 and, where possible, 72 h following injection of the drug. Plasma was separated immediately and stored at −20°C pending analysis. Plasma Adriamycin and adriamycinol concentrations were measured by a high performance liquid chromatography (HPLC) technique with fluorometric detection (Dobbs & James, 1987). Briefly, this involved extraction of Adriamycin and adriamycinol from plasma samples onto cartridges containing a C$^2$ bonded-silica material. Loaded cartridges were then introduced into the solvent stream of an HPLC system by an advanced automated sample processor (ASAP R; Varia Associated,

Correspondence: P.J. Johnson.
Received 12 July 1991; and in revised form 22 November 1991.

Walton-upon-Thames, UK). Peak heights were measured by a computing integrator and used to calculate peak height ratios for drug and metabolite. The detection limit for this method is 2 ng ml$^{-1}$. The necessary level of containment could not be provided for the safe extraction of samples obtained from patients known to be positive for the hepatitis B surface antigen (HBsAg) using this technique. Such samples were extracted by a chloroform:2-propanol (1:1) mixture as described by Andrews, Brenner, and Chou *et al.* (1984). Following evaporation of this solvent virological analysis indicated that these extracts could be handled safely. Extracted residues were redissolved in 150 µl of solvent and 50 µl injected into the HPLC system manually (detection limit 4–5 ng ml$^{-1}$). All other conditions for the assay were the same as for the former method.

*Pharmacokinetic analysis*

Plasma drug concentration-time data were fitted to both two and three compartment models using an iterative least squares regression programme with weighted, 1 y$^{-2}$ data (Yamaoka *et al.*, 1981; Johnston & Woollard, 1983). Minimisation of the residual sum of squares and Akaike's information criteria (Yamaoka *et al.*, 1978) were used to define the best choice of model to fit the data. The area under the plasma drug concentration-time curve (AUC) to 48 h was measured using the linear trapezoidal approximation for both Adriamycin and adriamycinol together with the AUC extrapolated to infinity, and the terminal half-life of Adriamycin.

*Assessment of response*

Response was assessed 2 to 3 weeks after the first injection of Adriamycin and then at 6-weekly intervals according to WHO guidelines (WHO handbook, 1979). A response was considered to have occurred when there was at least a 30% reduction in hepatomegaly as assessed clinically by distance below the costal margin or at least a 50% reduction in tumour diameter on serial ultrasound examination in patients with a solitary mass. In addition, a response was recorded if there was at least a 50% fall in the serum AFP level (where the pretreatment levels was greater than 250 ng ml$^{-1}$) provided that there was no contradictory evidence from the other two criteria.

*Statistical approach*

To assess the impact of the liver tests and pharmacokinetic parameters on the degree of myelosuppression we undertook an initial series of univariate analyses, followed by stepwise multiple regression analysis (BMDP: University of California, 1981). The independent influence of each of the liver tests, the AUC curve to 48 h (for Adriamycin and adriamycinol) and extended to infinity (Adriamycin only), and the terminal half-life (Adriamycin only) on the nadir values for haemoglobin, WCC and platelet count was examined. In all instances the white cell nadir was on day 14. The distributions of values of alkaline phosphatase, AST and platelet count were highly skewed, and the analysis was therefore undertaken after logarithmic transformation of this data. Differences in cumulative time-concentration curves were compared by applying Student's *t*-test at each individual time point, and patient survival curves were calculated using the Kaplan-Meier method.

The investigation protocol was passed by the Ethical Committee of King's College Hospital and all patients gave informed consent after being appraised of the possible risks involved.

### Results

Of the 16 hyperbilirubinaemic patients studied, five were unevaluable for response (one died within one week of starting treatment, and four overseas patients returned home where they could not be further traced). A response was documented in two patients. One of these died suddenly, 3

**Table I** Pre-treatment clinical and laboratory features of the 30 patients studied

| Patient | Age (years) | Sex | Nationality | Cirrhosis | AST (IU l$^{-1}$) | SAP (IU l$^{-1}$) | ALB (g l$^{-1}$) | Bilirubin (µmol l$^{-1}$) | Hb (g l$^{-1}$) | WCC ($\times 10^9$ l$^{-1}$) | Platelets ($\times 10^9$ l$^{-1}$) | HBsAg |
|---|---|---|---|---|---|---|---|---|---|---|---|---|
| 1 | 56 | M | UK | CAH | 34 | 114 | 37 | 36 | 13.4 | 2.2 | 62 | – |
| 2 | 67 | M | UK | ALD | 115 | 440 | 36 | 20 | 10.1 | 7.4 | 226 | – |
| 3 | 30 | M | India | NK | 66 | 143 | 41 | 15 | 13.5 | 8.7 | 213 | – |
| 4 | 50 | F | Saudi Arabia | NK | 47 | 183 | 29 | 34 | 12.8 | 7.9 | 152 | – |
| 5 | 72 | M | UK | CAH | 95 | 244 | 34 | 12 | 10.3 | 3.9 | 188 | + |
| 6 | 52 | M | Portugal | CRY | 101 | 163 | 35 | 37 | 12.5 | 6.6 | 94 | – |
| 7 | 71 | F | UK | NK | 145 | 393 | 32 | 22 | 10.8 | 6.8 | 499 | – |
| 8 | 76 | M | UK | CRY | 110 | 155 | 32 | 29 | 17.7 | 8.2 | 162 | – |
| 9 | 75 | M | Saudi Arabia | CRY | 180 | 393 | 22 | 25 | 9.8 | 6.1 | 280 | – |
| 10 | 24 | M | Ghana | CAH | 321 | 957 | 34 | 29 | 11.6 | 5.7 | 323 | – |
| 11 | 42 | M | Cambodia | CAH | 31 | 119 | 37 | 9 | 15.8 | 6.7 | 158 | + |
| 12 | 55 | M | Djibouti | CAH | 49 | 464 | 33 | 34 | 12.1 | 3.5 | 97 | – |
| 13 | 58 | M | UK | CRY | 56 | 277 | 31 | 17 | 10.5 | 15.8 | 327 | – |
| 14 | 33 | F | UK | – | 39 | 400 | 29 | 13 | 11.7 | 16.4 | 227 | – |
| 15 | 47 | F | Philippines | NK | 34 | 140 | 38 | 12 | 14.1 | 10.3 | 341 | – |
| 16 | 36 | F | UK | – | 73 | 348 | 41 | 5 | 13.5 | 11.6 | 368 | – |
| 17 | 74 | M | UK | ALD | 47 | 160 | 31 | 23 | 13.0 | 8.0 | 168 | – |
| 18 | 56 | M | Burma | CRY | 64 | 116 | 43 | 15 | 13.0 | 10.8 | 284 | – |
| 19 | 49 | M | Italy | CRY | 57 | 290 | 40 | 13 | 15.3 | 5.3 | | – |
| 20 | 54 | M | India | CAH | 119 | 243 | 39 | 9 | 15.6 | 6.4 | 283 | – |
| 21 | 49 | M | Sicily | CRY | 103 | 192 | 27 | 19 | 11.6 | 6.3 | 119 | – |
| 22 | 31 | F | UK | – | 41 | 527 | 38 | 12 | 16.5 | 11.1 | 246 | – |
| 23 | 69 | M | UK | NK | 59 | 213 | 35 | 13 | 9.1 | 4.4 | 246 | – |
| 24 | 60 | M | UK | ALD | 64 | 272 | 29 | 31 | 12.5 | 5.0 | 100 | – |
| 25 | 20 | F | UK | – | 119 | 312 | 33 | 14 | 9.7 | 6.9 | 439 | – |
| 26 | 69 | F | UK | CAH | 149 | 648 | 31 | 28 | 9.5 | 8.1 | 425 | – |
| 27 | 43 | F | Kuwait | – | 136 | 335 | 36 | 9 | 10.0 | 3.9 | 79 | – |
| 28 | 51 | F | Ghana | NK | 156 | 975 | 25 | 20 | 9.5 | 12.0 | 66 | + |
| 29 | 52 | M | Hong Kong | NK | 57 | 228 | 30 | 37 | 14.2 | 3.3 | 105 | + |
| 30 | 63 | M | Egypt | ALD | 284 | 178 | 24 | 32 | 10.1 | 4.2 | 202 | – |

CAH = chronic active hepatitis, ALD = alcoholic liver disease, CRY = cryptogenic, NK = not known, SAP = serum alkaline phosphatase, ALB = albumin.

**Figure 1** Comparison of terminal half life and area under the concentration-time curves (at 48 h), for Adriamycin and adriamycinol in relation to hyperbilirubinaemia. The mean values are greater for each parameter in the hyperbilirubinaemic patients and this achieves statistical significance ($P < 0.01$) for Adriamycin and adriamycinol using Students $t$-test, but just fails to reach statistical significance if the data is logarithmically transformed.

months after starting treatment, following a variceal hemorrhage related to the underlying cirrhosis. Two further patients completed a full course of Adriamycin (total dose = 550 mg m$^{-2}$) and there was no evidence of tumour progression at 12 months. The remaining seven patients showed tumour progression as documented radiologically and/or by serial AFP estimations.

Three patients with a normal serum bilirubin concentration were unevaluable (one underwent liver transplantation at 3 weeks and two were lost to follow-up overseas). One of the 11 evaluable patients with a normal bilirubin, underwent a response, and three remain well at 6 months with no tumour progression and seven had progressive disease. Median survival time (from diagnosis) for those with hyperbilirubinaemia was 4 months (range 1 to 19 months), and for those with normal bilirubin 4 months (range 1 to 11 months). There was no significant difference in the Kaplan-Meier survival curves for the two groups.

### *Pharmacokinetics*

In 22 of the 30 patients a complete pharmacokinetic study was undertaken, and in four of these this was repeated during the second course. The remainder either declined to enter this part of the study, could not remain in hospital for the required period of time, or could not have an adequate venous access established. In all instances, the Adriamycin drug-concentration time curve was fitted best by a three compartment model. The AUC curve to 48 h and to infinity was significantly greater in hyperbilirubinaemic patients for both Adriamycin and adriamycinol (Figure 1), than in those with a normal serum bilirubin concentration. When drug concentration time curves were calculated by taking the mean (log) drug-concentration at each time point, the curve was significantly higher in the hyperbilirubinaemic patients (Figures 2a and b).

The terminal half-life of Adriamycin ranged from 11–75 h and was not related to serum bilirubin level or any other of the standard liver tests.

### *Toxicity*

For the series as a whole, the nadir for the haemoglobin and the white cell count (WCC) was consistently on day 14 and for platelets on day 7, although the fall in white cell count was far more profound than either of the other two (Figure 3). Despite this the white cell count had risen to more than $3 \times 10^9\, l^{-1}$ in all but four of the 21 patients in whom it could be measured by day 21, and in one of these the count had

**Figure 2** Comparison of (a) log Adriamycin concentration-time curves (ng ml$^{-1}$ h$^{-1}$) for patients with serum bilirubin concentration above (○) and below (●) 17 μmol l$^{-1}$ and (b) log adriamycinol concentration-time curves for patients with serum bilirubin levels above (○) and below 17 μmol l$^{-1}$ (●). Results are presented as mean plus or minus one standard deviation, * = $P < 0.01$, ** = $P < 0.001$.

been $2.2 \times 10^9\, l^{-1}$ at the time of treatment. There was a significant linear correlation between serum bilirubin level and white cell count at days 7 ($r = -0.6$, $P < 0.01$) and 14 ($r = -0.603$, $P < 0.01$) (Figure 4), and the white cell count was significantly lower in the hyperbilirubinaemic patients on both days 7 and 14. No correlation was detected with any other liver test or pharmacokinetic parameter on day 7 and 14, either using univariate or multivariate analysis.

The degree of thrombocytopenia also correlated with serum bilirubin before logarithmic transformation of the platelet count but when the latter was normalised, the only

**Figure 3** Changes in haemoglobin concentration, white cell and platelet counts with time following the standard bolus dose of 60 mg m$^{-2}$ of Adriamycin given intravenously.

**Figure 4** Relation of white cell nadir (day 14) to serum bilirubin concentration.

positive correlation was with log of serum alkaline phosphatase ($r = 0.65$, $P < 0.01$). A single patient (Patient record 4, Table I) developed septicaemia whilst neutropenic (WCC = $0.6 \times 10^9$, day 10) but this responded rapidly to appropriate antibiotic therapy. Otherwise side-effects were remarkably uncommon. All patients developed some degree of alopecia. One patient developed severe, and one mild mucositis. Three had rigors, lasting less than one hour, at 2, 4 and 10 h after the injection. Seven patients complained of nausea on the morning following drug administration and this was associated with vomiting in four.

Of the 100 consecutive patients with HCC surveyed since 1986, the serum bilirubin was above 40 μmol $l^{-1}$ in 29%, between 18 and 39 μmol $l^{-1}$ in 39% and within the reference range in 39%.

### Discussion

More than 60% of our patients with HCC were hyperbilirubinaemic at presentation and would, therefore, be candidates for Adriamycin dosage reduction using conventional regimens. Although we show here that it may be safe to administer a full dose of Adriamycin to those HCC patients with mild hyperbilirubinaemia, the number of evaluable patients was too small for us to make a confident statement as to whether this regimen might influence response or survival rate.

Nonetheless, the response rate of only 18% was disappoint ingly low and similar to the figure obtained in a previous study of patients in whom the Adriamycin dose was decreased in line with the degree of hyperbilirubinaemia (Johnson *et al.*, 1986).

In our previous study (Johnson *et al.*, 1986) the response rate was higher (45%) in those with normal bilirubin levels suggesting that hyperbilirubinaemia (or a factor closely associated with it) is a genuine adverse factor indicating a decreased likelihood of response to Adriamycin and that the overall response rate, in a particular group of patients, will be related to the distribution of bilirubin levels. This observation was not confirmed in the present study although the number of patients in the trial is considerably smaller, and this reflects the trend that response rates quoted in more recent studies are lower than the initial reports. It is difficult to compare survival figures with early series since these are influenced to a considerable extent by the time of diagnosis of the tumour, and with the advent of screening programmes for patients with cirrhosis and much more sensitive ultrasound techniques for detection, diagnosis was being made significantly earlier, with consequent apparent prolongation of survival.

The liver is the major site of metabolism of Adriamycin and approximately 40% of the drug is excreted via the biliary tract (Riggs, 1977). Early studies (Benjamin *et al.*, 1981) in hyperbilirubinaemic patients with metastatic liver cancer receiving intermittent bolus therapy demonstrated a delayed clearance of Adriamycin. This formed a rational explanation for the clinical observation that some of these patients developed severe toxicity, particularly myelosuppression and mucositis. It was on the basis of these observations that the current dose reduction schedule in the presence of hyperbilirubinaemia has been applied to patients with secondary liver cancer. There have, however, been some theoretical reasons suggested why this may not necessarily apply to patients with primary liver cancer. Thus Chleblowski *et al.* (1981) have suggested that in the patient with HCC, the tumour tissue may retain some effective drug-metabolising capacity, whereas in metastatic liver disease the tumour replaces functioning liver tissue. Similarly Chan *et al.* (1980), on the basis of pharmacological studies showing little difference between HCC patients with disturbed liver tests and other patients with normal liver function, have also suggested that a 'more rigorous dose regimen' might be advantageous in the treatment of HCC patients, despite some degree of hepatic dysfunction.

Our data show that both views may be correct to varying degrees. Thus the degree of leucopenia was inversely related to the height of the serum bilirubin concentration and Figure 4 suggests that administration of the standard Adriamycin dose (60 mg $m^{-2}$) to patients with bilirubin concentrations higher than 40 μmol $l^{-1}$ would be most hazardous. Indeed, the white cell count in the present patients may well have been lower between days 7 and 14, and the degree of neutropenia greater than indicated by the total white cell count. However, with the exception of one patient who developed septicemia, the patients tolerated the regimen and any associated myelosuppression well, and unlike the experience of Benjamin *et al.* (1981), mucositis was uncommon. Whilst the relevance of this clinically is limited by the lack of improvement in response rate, the finding may still be valuable in respect of the use of Adriamycin given by other approaches such as intra-arterially, where is efficacy appears greater (Patt *et al.*, 1987), or in combination with other drugs.

Ballet *et al.* (1984) have reported a diminished hepatic extraction of Adriamycin with values of less than 0.1 in five HCC patients with cirrhosis and concluded that there is an impairment of cellular transport in HCC patients even in the absence of severe hepatic dysfunction. However these results have been criticised (Kaye *et al.*, 1985) on the grounds that no distinction between Adriamycin and adriamycinol was made and the timing of the sampling, and they are difficult to reconcile with the normal values for clearance reported by Chan *et al.* (1980) and referred to above. Much higher extraction ratios were reported by Garnick *et al.* (1979) in cancer patients with abnormal liver function tests, but these patients did not have cirrhosis or HCC.

The extent to which toxicity is related to the pharmacokinetics of Adriamycin remains controversial (Kaye *et al.* (1985). Previous studies which have concentrated on comparison of pharmacokinetic parameters between patients with normal and compromised liver function have suggested that clearance of both Adriamycin and its metabolites was delayed in patients with hyperbilirubinaemia (Benjamin *et al.* (1981) but later work using HPLC techniques found little difference in Adriamycin disposition though formation and excretion of adriamycinol was invariably delayed (Chan *et al.*, 1980). Our data support the latter finding in that the AUC for adriamycinol to 48 h was significantly greater in those with hyperbilirubinaemia and the mean concentration-time curve was also higher.

We could detect no relationship between the degree of toxicity and any parameters including AUC and terminal half-life, for either Adriamycin or adriamycinol. It is likely, however, that the failure to detect any positive correlations is a reflection of the small number of patients with complete data and the narrow range of white cell nadirs seen in the present patients irrespective of the serum bilirubin level (1.7 to $4.2 \times 10^9$ $l^{-1}$).

Myers (1982) concluded that 'a convincing quantitative relationship between liver function abnormalities and impaired Adriamycin clearance has not been established'. However, the present study strongly suggests that 'liver function', at least as reflected by the serum bilirubin concentration, does indeed influence Adriamycin clearance. Our results imply that this is reflected in increased toxicity; direct statistical confirmation of this contention would almost certainly require very large numbers of patients.

### References

ANDREWS, P.A., BRENNER, D.E., CHOU, F.T.E., KUBO, M. & BACHUR, N.R. (1984). Facile and definitive determination of human adriamycin and duanorubicin metabolites by high pressure liquid chromatography. *Drug Metabol. Dispos.*, **8**, 152.

BALLET, F., BARBARE, J.C. & POUPON, R. (1984). Hepatic extraction of adriamycin in patients with hepatocellular carcinoma. *Eur. J. Clin. Oncol.*, **20**, 761.

BENJAMIN, R.S., WIERNIK, P.H. & BACHUR, N.R. (1981). Adriamycin chemotherapy - efficacy, safety, and pharmacological basis of an intermittent single high-dose schedule. *Cancer*, **48**, 1088.

BMDP: *Statistical Software*. Berkely: University of California Press 1981.

CHAN, K.K., CHLEBOWSKI, R.T., TONG, M., CHEN, G.H.S., GROSS, J.F. & SATEMAN, J.R. (1980). Clinical pharmacokinetics of adriamycin in hepatoma patients with cirrhosis. *Cancer Res.*, **40**, 1263.

CHLEBOWSKI, R.T., CHAN, K.K., TONG, M.J., WEINER, J.M., RYDEN, V.M.J. & BATEMAN, J.R. (1981). Adriamycin and Methyl-CCNU combination therapy in hepatocellular carcinoma: Clinical and pharmacokinetic aspects. *Cancer*, **48**, 1088.

DOBBS, N.A. & JAMES, C.A. (1987). Estimations of doxorubicin and doxorubicinol by high pressure liquid chromatography and advanced automated sample processor. *J. Chromatog.*, **420**, 814.

GARNICK, M.B., ENSMINGER, W.D. & ISRAEL, M.A. (1979). Clinical-pharmacological evaluation of hepatic arterial infusion of Adriamycin. *Cancer Res.*, **39**, 4105.

JOHNSON, P.J., WILLIAMS, R., THOMAS, H. & others (1978). Induction of remission in hepatocellular carcinoma with doxorubicin. *Lancet*, **i**, 1006.

JOHNSON, P.J., ALEXOPOULOS, A.T., JOHNSON, R.D. & WILLIAMS, R. (1986). Significance of serum bilirubin in response of hepatocellular carcinoma to doxorubicin. *J. Hepatol.*, **3**, 149.

JOHNSTON, A. & WOOLLARD, R.C. (1983). Stripe: An interactive computer program for the analysis of drug pharmacokinetics. *J. Pharmacol. Meth.*, **9**, 193.

KAYE, S.B., CUMMINGS, J. & KERR, D.J. (1985). How much does liver disease effect the pharmacokinetics of adriamycin? *Eur. J. Clin. Oncol.*, **21**, 893.

MYERS, C.E. (1982). Anthracyclines. In *Pharmacologic Principals of Cancer Treatment*. Chabner, B.A. (ed.), W.B. Saunders: Philadelphia, pp. 426–448.

OLWENY, C.L.M., TOYA, T., KATONGOLE-MBIDDE, E., MUGERWA, J.A., KYALWAZI, S.K. & COHEN, H. (1975). Treatment of hepatocellular carcinoma with doxorubicin. *Cancer*, **36**, 1250.

PATT, Y.Z., CHARNSANGAVEJ, C., SOSKI, M. & MAVLIGHT, G.M. (1987). Regional arterial therapy in the management of primary liver neoplasms. In *Liver Cancer*, Bottino, J.C., Opfell, R.W. & Muggia, F.M. (eds), Marinus Nijhoff Publishing, Boston.

PETERS, R.L. (1976). Pathology of hepatocellular carcinoma. In *Hepatocellular Carcinoma*, Okuda, K. & Peters, R.L. (eds), John Wiley & Sons, New York.

RIGGS, C.E. (1977). Biliary disposition of adriamycin. *Clin. Pharm. Therap.*, **22**, 234.

VOGEL, C.L., BAYLEY, A.C., BROOKER, R.J., ANTHONY, P.P., PATH, M.R.C. & ZIEGLER, J.L. (1977). A Phase II study of adriamycin (NSC 123127) in patients with hepatocellular carcinoma from Zambia and the United States. *Cancer*, **39**, 1923.

WHO (1979). *Handbook for Reporting Results of Cancer Treatment*. WHO: Geneva.

YAMAOKA, K., NAGAGAWA, T. & UNO, T. (1978). Application of Akaike's information criterion (AIC) in the evaluation of linear pharmacokinetic equations. *J. Pharm. Biopharm.*, **6**, 165.

YAMAOKA, K., TANIGAWARA, Y., NAKAGAWA, T. & UNO, T.A. (1981). A pharmacokinetic analysis program (multi) for microcomputer. *J. Pharm. Dynam.*, **4**, 879.